# A Survey of Public Health Failures During COVID-19

**DOI:** 10.7759/cureus.32437

**Published:** 2022-12-12

**Authors:** Beatriz C Dominquez, Amanda Hernandez, Alexnys Fernandez-Pacheco, Lauren Taylor, Payal Kahar, Deepesh Khanna

**Affiliations:** 1 Foundational Sciences, Dr. Kiran C. Patel College of Osteopathic Medicine Nova Southeastern University, Clearwater, USA; 2 Foundational Sciences, Nova Southeastern University Dr. Kiran C. Patel College of Osteopathic Medicine, Clearwater, USA; 3 Osteopathic Medicine, Nova Southeastern University Dr. Kiran C. Patel College of Osteopathic Medicine, Fort Lauderdale, USA; 4 Department of Health Sciences, Florida Gulf Coast University, Fort Myers, USA

**Keywords:** quarantine, covid-19 vaccine hesitancy, travel screening, hand hygiene, coronavirus quarantine, lockdown, social distancing, mask mandates, coronavirus infection, covid-19

## Abstract

The prolonged coronavirus disease 2019 (COVID-19) pandemic has raised concerns about the failures in the public health measures used to manage the spread of this deadly virus. This review focuses its attention on research papers that at their core highlight the individual public health measures instituted by organizations, institutions, and the government of the United States (US) since the start of the COVID-19 pandemic and that were published in 2019 to 2022. Together, these sources help paint a well-rounded view of the US management of this pandemic so that conclusions may be drawn from mistakes that were made and this country may respond better in the future to such situations. This paper is unique because it highlights the areas where improvement is needed, whereas other published work describes the measures taken and how they were carried out, not the failures, which leaves a gap in the literature that this paper hopes to fill. Through a deep dive into public health measures, seven areas in which improvements could be made were pinpointed by the authors. Such measures included mask mandates, social distancing, lockdown/quarantine, hand hygiene, COVID-19 testing, travel screening, and vaccine hesitancy. In exploring each measure, a discussion was carried out about its benefits and shortcomings in alleviating the ramifications of a global pandemic. In addition to the poor supply chain for critical products like personal protective equipment (PPE), the miscommunication between states and federal policies did not allow for the entirety of the US to respond cohesively in the face of the COVID-19 pandemic. This general review is crucial to know what is working and what needs to be changed to increase the benefits provided to the population.

## Introduction and background

The coronavirus disease 2019 (COVID-19) virus, also known as severe acute respiratory syndrome coronavirus (SARS-CoV-2), was identified in late 2019 and quickly became a super spreader virus across the world. COVID-19 is an incredibly fast-spreading virus, spreading through respiratory droplets. The disease may not show symptoms right away. This contributes to how vastly spread it became and why it became a pandemic. Most of the population will experience mild to moderate symptoms but will recover without issues [1]. Those with weaker immune systems, such as the elderly, pregnant women, cardiovascular disease patients, diabetics, and cancer are more likely to experience serious respiratory symptoms [1-4]. To date, there has been a total of 34 million cases and 622,000 deaths [5]. While there has not been a pandemic of this extent in quite some time, similar precautions were taken during the Spanish Flu of 1918 and when the H1N1 strain hit the US. During the Spanish Flu, people were quarantined while with the H1N1 strain mostly handwashing and avoiding contact were encouraged. The public health measures taken back then were vastly different from the ones put in place during COVID-19 due to the expansive spread. This review's objective is to analyze the failures of public health measures in the US and to conceptualize how to move forward. Specifically, failures in mask implementation, social distancing, and lockdowns. These public health measures were considered a failure in preventing the rapid spread of COVID-19. Other failures that were discussed included travel screenings, rapid testing inefficiency, and people unwilling to vaccinate.

## Review

Methods

A review of the failures of public health measures in the US during the COVID-19 pandemic was performed using online sources such as PubMed, Google Scholar, and the Centers for Diseases Control and Prevention (CDC) website. Within the research, the main search words used to find articles were COVID-19, public health measures, mask mandates, lockdown, and vaccinations. The articles selected were about the main safety measures implemented to prevent the spread of COVID-19, as well as articles showing COVID-19 progression throughout different states. Articles were excluded in which public health measures related to COVID-19 were not mentioned. Specifically, the main reason for excluding 2106 articles in the first pass, was no mention of COVID-19 in the title or abstract of the articles. The CDC, with the World Health Organization (WHO), was used for data regarding the number of cases, deaths, and modes of contamination. Articles from before 2019 were excluded. The study was conducted according to the latest Preferred Reporting Items for Systematic Reviews and Meta-Analyses (PRISMA) guidelines. The inclusion criteria for the 61 articles included in the review consisted of articles specific to the US population, published after 2019, mentioning a specific public health measure taken during the pandemic, and relevant to COVID-19.

**Figure 1:**
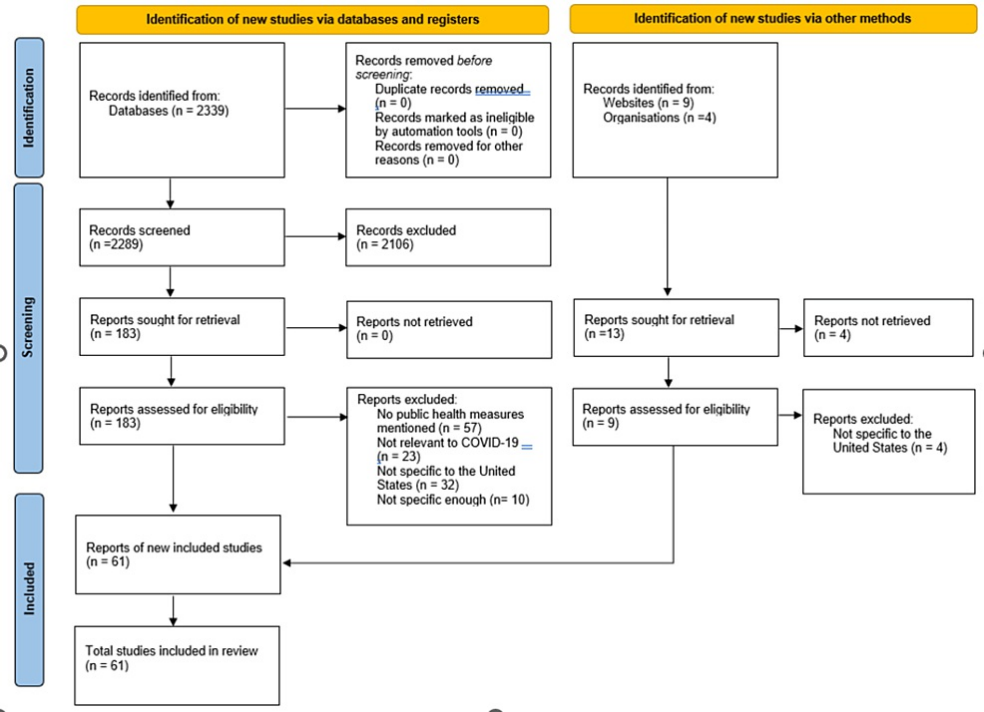
PRISMA Flow Diagram

Discussion

Masks

The use of masks to prevent the spread of the COVID-19 virus was widely recognized across the US. Mask mandates came into play at varying times throughout the pandemic for each state and faced criticism on both sides of the spectrum. Masks were majorly used indoors in most states, while few states required their use in outdoor environments. Masks have a 99.98%, 97.14%, and 95.15% efficacy for N95, surgical, and homemade masks, respectively in preventing the spread of avian influenza [6]. The avian influenza was used as a comparison because of the similarity in size with the COVID-19 virus. A study on the spread of the viral particles of COVID-19 was performed, using a mannequin, which demonstrated a decreased transmission of over 50% with cotton and surgical masks and of 80-90% reduction with N95 masks, as shown in Figure 1 [7]. Across multiple studies, the risk of infection with the COVID-19 virus was reduced using masks, whether N95, surgical, or cotton masks [8]. According to the CDC, the guidelines for mask wearing were to make sure they are well-fitted, and the correct type of mask that should prevent the aerosolized matter from penetrating and worn indoors and outdoors if in contact with other people [9].

**Figure 2:**
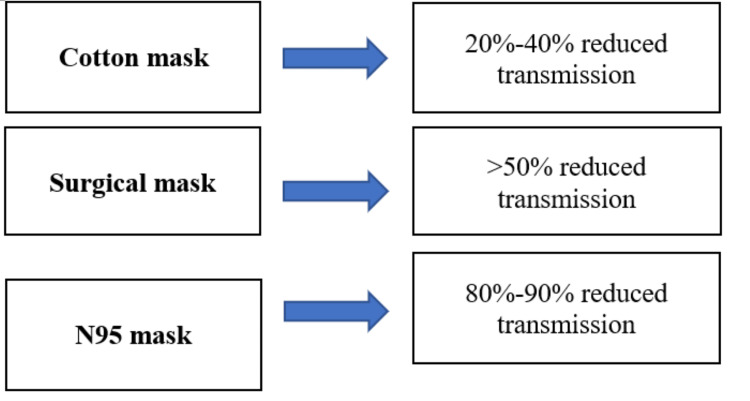
Mask Type and Reduced Transmission of COVID-19

Public health failed with the implementation of mask mandates because it was not implemented in a timely and uniform matter across the US. The states made the individual decisions of when to require the mask mandates and the specific regulations surrounding it. Some states failed to implement any mandates at all and left the local government to decide for themselves. States such as New York and Washington took on universal mask mandate policies, which showed a decrease in the spread of COVID-19 [10]. These universal mask mandates require residents of the state to wear masks whether indoor or outdoor areas regardless of close contact with other individuals. While masks proved to be an efficacious method to prevent COVID-19 transmission, there were many states which still had not implemented this policy on their residents. By September 2020, there were still 14 states which had not implemented any mask mandate orders [10]. To date, there are still 11 states which have never implemented mask mandates all over their population [11]. This can lead to a higher transmission rate among those states and prolong the spread of the virus. States such as Arizona, Utah, and Rhode Island had the highest cases per 100,000 people across the US [12]. All the previously mentioned states failed to issue a state-mandated mask order which led to a high increase in cases per capita [10]. While states such as Maryland, Oregon, and Pennsylvania quickly carried out the state-mandated mask order and ended up with some of the lowest averages in total cases [10].

Issues also became apparent with the type of masks used. The CDC recommended the use of N95 masks for healthcare workers, as they would have the highest exposure to the virus [9]. However, many civilians purchased these masks as well which led to a shortage in the healthcare field. This was a barrier many hospitals had to overcome by using surgical masks. The lack of information about the types of masks to be used had a substantial impact on the population because even though cloth and homemade masks were shown to be efficacious, the way they were fitted and used had a much larger impact. Surgical masks, which are meant for one-time use, were being reused by the public. These masks, when wet or dirty from constant reuse reduce the efficacy of preventing transmission [9]. Homemade and cloth masks were not always sized appropriately, and individuals had large gaps around the side of the masks or loose ear loops, which is directly contraindicated by the CDC [9].

Lastly, there was a shortage of personal protective equipment, specifically face masks, that the US was unprepared for when the pandemic hit. Increased use of surgical and N95 masks led to dwindling resources because the demand far exceeded the supply available [13]. Hospitals found themselves having to use reusable equipment when lacking the one-use equipment, they are usually afforded. Outside of the healthcare setting, there was a lack of masks provided to the public. While a state may have put in place a mask mandate, that does not inherently mean that a population has constant access to adequate masks. Only a few businesses were able to provide masks to their patrons, and this lack of availability led to individuals using inadequate replacements for masks such as neck gaiters, face shields, and masks with vents [14]. 

The timely and uniform execution of mask mandates, adequate supply, and properly informing the public are major considerations for future purposes. Several states showed much better results and decreased COVID-19 cases when those guidelines were followed [14]. Furthermore, healthcare workers could continue to perform their daily job duties without feeling at risk or having to quarantine themselves after a possible exposure. Leading more workers in the field to take care of those who become ill. Data showed that mask use is an efficacious way to prevent transmission of COVID-19 and proper application and supply can lead to a decrease in cases in the future [14].

COVID-19 Testing

During the past year and a half, diagnostic testing has been critical for quickly and accurately identifying individuals infected with COVID-19 and battling the spread of the virus. Identifying failures within the diagnostic methods used during this pandemic can be especially useful in helping us respond to future pandemics more efficiently.

Throughout this pandemic, several types of diagnostic tests were employed to determine who had contracted COVID-19 and was fighting a current infection. The first type of testing most used was a polymerase chain reaction (PCR) test done on samples acquired on a swab from the nasal passage. PCR tests require three reagent kits and need to be analyzed in a lab with specific lab equipment making the process lengthier [15]. Ultimately, when compared to other tests in the market, PCR is the most sensitive diagnostic test and therefore is more accurate in diagnosing patients carrying COVID-19 [15]. The second most common type of testing administered is an antigen test on blood samples. For this testing method, results can be attained in 15 minutes as it does not need to be evaluated in a lab but can be less sensitive when compared to PCR testing [15]. The decrease in accuracy in antigen tests can be attributed to the fact that such a test must be done on a fresh sample and delaying testing by a couple of days can lead to antigen destruction and result in a false-negative result [15].

One major failure in COVID-19 diagnostic testing was the depletion of testing kits. At the start of the pandemic, the PCR method was the test used most in the US because serologic testing kits had not been mass-produced and dispersed yet. As the virus continued to spread and more individuals got sick, the demand for testing increased exponentially while the supply decreased due to the lack of available materials to conduct the PCR tests. Several months into the pandemic the US Strategic National Stockpile (SNS), which stores medical equipment for emergency events like this one, was running low due to the worsening situations in hospitals across the country [16]. As mentioned previously, PCR tests require the use of three reagents and with the global shutdown, such materials were hard to accumulate quickly enough to restore the SNS. The global shutdown was critical because the US relies on imports from other countries to supply the healthcare sector with many minerals being exclusively sourced from outside the US [16]. This resulted in the early closure of testing sites due to a limited number of testing kits with many individuals being turned away. This was a huge issue because not diagnosing in time meant that people continued to spread the virus in their communities and to everyone they encountered. Later, this issue was alleviated by the production of serologic tests that did not use such materials and that could produce results within minutes, but by then a lot of damage had been done.

In conjunction with the low availability of testing kits, there was also a lack of access to testing sites. When testing became available, testing sites were set up by government agencies throughout counties across the US in places like stadiums and churches and many others. The location of such testing sites highlights a huge disparity in the access that different racial and ethnic groups have to medical services. As detailed in a recent study done on COVID-19 testing site distribution in New York City, data show that African American and Latino communities have fewer sites in their zip codes than traditionally white communities [17]. Health disparities like these are particularly harmful because data also show that African American and Latino communities account for nearly 20 percent more positive COVID-19 results than white communities [17]. When considering the population density and distribution in a dense city like New York City, the lack of access to testing sites by minority groups can lead to debilitating consequences.

Another prevalent issue with COVID-19 testing was the long wait times for testing results. With the huge demand for testing, especially in highly populated cities, the turnaround time for results was greatly affected. Results would take anywhere from 24 to 72 hours which then took one to five days to be relayed to the patient [18]. Many factors affected this delay which included low staffing in the lab at one time due to CDC regulations and depletion of the raw materials to carry the tests out [18]. Simply put, the demand for testing results was too high to be met promptly. Long turnaround times meant that individuals could be carrying the virus and going to work or their communities and spreading it.

During the COVID-19 pandemic, there were many issues in the way testing methods were developed and employed to battle the quick spread of the virus. To be better prepared, the US can work on building a larger stockpile of raw materials for the tests discussed above, and in doing so, be sure that there are enough kits to diagnose individuals and stop the spread of disease. It would be beneficial to work on having these materials sourced in the US as opposed to being out of the country so that the US is less affected by another global shutdown. Better policies can be established to ensure that racial background or the zip code someone lives in is not a determinant of their access to medical services, such as testing. These are just a few things that if changed can make a world of difference in response to any future pandemic. 

Social Distancing

One of the main public health measures used to contain the spread of any contagious disease is social distancing. Theoretically, the fewer people one is in contact with, the spread of disease decreases, and the chance of getting sick is lower. In a study comparing the COVID-19 incidence and mortality rates of 3,054 US counties during the first wave of the pandemic, it was determined that increased social distancing measures decreased incidence by 26% and mortality by 31% [19]. This is not a new measure; social distancing has been used for hundreds of years when diseases have popped up across the world. For example, in the 2009 H1N1 pandemic modeling studies showed that social distancing, especially in workplaces, led to a reduction in cases [20]. Therefore, it comes as no surprise that this measure is at the top of the list when confronting the current COVID-19 pandemic. Many failures can be assessed regarding the US implementation of social distancing and how improvements could have been made to ensure maximum benefit during this pandemic.

The physical parameters of social distancing have been widely studied to determine what is a safe distance that ensures low to no transmission of the virus. One of these studies assesses the distance traveled by viral particles during regular breathing, sneezing, and coughing to determine the appropriate social distance to avoid the zone of viral exposure [21]. This study found that sneezing and coughing can cause viral particles to be transported long distances in part due to the velocity at which particles are ejected in such actions [21]. This is without taking into consideration external factors like temperature or ventilation which could aid in increasing the distance traveled by the particles [21]. The data determined that the distance of two meters suggested by WHO is not enough to avoid the exposure zone and that it needs to be at least five meters [21]. Studies analyzing the physical travel of COVID particles were not carried out in time to prevent exposure and even when they adhered to the two meters, or six feet rule was already well established.

When evaluating the US use of social distancing measures in response to COVID-19, the question of timing is always discussed. Were social distancing measures established at the appropriate times or would it have been beneficial from an earlier start? This very question has been statistically evaluated through a longitudinal study of 37 countries done after the first wave of the pandemic [22]. The study maps when each country implemented school closures and banned mass public gatherings in comparison to their cumulative mortality from COVID-19 [22]. Through the statistical evidence presented in this study, it can be observed that countries that enforced earlier social distancing measures had lower mortality when compared to countries that waited longer to implement such measures [22]. The study also discusses the effects of an earlier start to social distancing by mathematically mapping the estimated cumulative COVID-19 mortality if each country had started just one week earlier than when they did [22]. It was estimated that by doing so each country could have decreased such numbers by about 44 % [22]. This positive correlation between an earlier start to social distancing and the decrease in cumulative mortality cannot be overlooked when evaluating the importance of this public health measure and the US's failure to institute it sooner.

Another facet of social distancing that needs to be discussed is how it differed from state to state. One of the key issues at the start of the COVID-19 pandemic was the differing mandates proposed from state to state which often led to different states being at various stages of pandemic management. In a state-level analysis done with data collected from the start of the COVID-19 pandemic, it was discovered that states that had stay-at-home orders and public gathering bans in place by the time they reach their 500th COVID case had lower transmission rates and longer case doubling times [23]. The transmission rates were measured by analyzing the average effective reproductive number for each state the week after they reached their 500th case on record [23]. Another study conducted analyzed data from 27 US states to determine the connection between overall mortality and maximum mortality rate and population density [24]. It showed that states with higher population density recorded higher overall mortality and higher maximum mortality rates [24]. This connection suggests that such states with higher population densities have increased frequency of social interactions which directly affect the transmission of the COVID-19 virus and therefore mortality rates from the said virus. The lack of a cohesive social distancing policy across all 50 states could have been a huge factor in decreasing or even preventing the high mortality rates seen throughout the US. States with a greater concentration of population could have benefited from equal enforcement of such policies.

With retroactive analysis of data gathered from the start of the COVID-19 pandemic and peak points during the last year, there were failures in instituting adequate social distancing measures. Discovering the safe social distance needed to avoid exposure took some time and therefore not established in public settings. Another big failure was the timing of when these measures were enforced. As discussed above, based on data gathered from different countries, an earlier start time has been mathematically proven to lead to decreased overall mortality. A final failure in implementing social distancing measures was the lack of cohesive policy enforced by all states in the US. Various policies from state to state led to varying degrees of enforcement and in the long run compliance by the population which ended up affecting some states more negatively than others.

Vaccine Hesitancy

For months, the primary way to combat the spread of COVID-19 was through non-pharmaceutical measures, such as lockdowns and mask mandates [25]. These procedures were effective in preventing the spread when implemented purposefully and on a national level. The approval of three different vaccines for emergency use in the US, the Pfizer, Moderna, and Johnson & Johnson vaccines, has allowed the focus to change from non-pharmaceutical measures to vaccine rollout and implementation. Though many people have been given the vaccine, many people have still not received it.

Vaccine hesitancy found in the US can be attributed to the three C model, which is composed of the three categories of convenience, confidence, and complacency [26]. These categories measure the accessibility and ability of the public to get the vaccine, the credibility of the safety and effectiveness of the vaccine, and the public’s perception of the need to receive a vaccine for preventable illness, respectively. Though it is not guaranteed, it was found that when vaccines are offered in more accessible locations, such as schools, and are shown to be financially beneficial, the public is more likely to get those vaccines [27]. The confidence of the public, or concerns about efficacy and potential side effects, was found to be the most valued reason behind the reluctance to get the COVID-19 vaccine [28]. It was recently shown to be of great importance when the Johnson & Johnson vaccine was cited as potentially associated with blood clots [29]. Though later assured to have a very weak correlation, this setback likely caused the public to rethink their trust in any of the vaccines against COVID-19. Complacency is also shown to have a positive relationship with vaccine hesitancy. Because many people who are younger or of lower socioeconomic status have a lower perception of risk and disease severity, they are less likely to engage in COVID-19 vaccine uptake [30].

This three-C model is furthered with the addition of two more categories, communication, and context [27]. These categories convey the importance of access to valid information and understanding cultural factors. Many of the concerns about potential side effects or the effectiveness of the COVID-19 vaccine are caused by misinformation given by the mainstream media. This misinformation has been able to spread quickly because of the volume of information being conveyed within the media and the rapid advancements of the COVID-19 pandemic [27]. The misleading information given to the public can create confusion and distrust, which emphasizes the need for an educational initiative focused on public health as well as timely information communicated at a community level [25].

An increase in vaccine hesitancy within the US has also been correlated with communities of ethnic and racial minorities [31]. A review of multiple studies showed that the largest minorities in the US, Hispanic and African Americans, were more likely to be hesitant in receiving the vaccine due to reasoning associated with sociodemographic characteristics, like income and education, as well as medical mistrust, beliefs about the vaccine, and exposure to misinformation [31]. Another study revealed that African Americans had the highest prevalence of vaccine hesitancy, and the likelihood of vaccine hesitancy increased as the level of education, household income, and fear of infection decreased [32]. Gender was also found to be less of a determinant of hesitancy to get a COVID-19 vaccine, but the reasoning for hesitancy was found to be different when considering gender; women were more likely to have hesitancy due to circumspection, whereas hesitancy in men could be attributed to complacency [33].

To increase vaccination rates in the US, some corporations and state governments have started to offer monetary motivation as well as the promise of other material rewards. States, such as Ohio, North Carolina, and Michigan, have offered their citizens a drawing in a lottery with a chance at winning a sum of a million dollars or more in exchange for getting a vaccination [34]. Some city officials, like the ones in Memphis, Tennessee, have even offered the chance of winning a new vehicle for each newly vaccinated individual [34]. Grocery stores, such as Aldi and Publix, and transportation services, like Amtrak and American Airlines, are offering their employees varying levels of compensation to show proof of getting a COVID-19 vaccine [35]. Even though rewarding incentives are being offered, vaccination rates are still not rising at the desired rate. Some corporations have decided to require their employees to receive the COVID-19 vaccine; major businesses, like Goldman Sachs, Google, and The Walt Disney Company, are implementing a vaccine mandate that requires all their employees to have it unless there are legitimate medical or religious concerns [36].

Lockdown & Quarantine

As the COVID-19 pandemic began its initial spread, many countries were not prepared with a plan on how to contain the virus since there had not been a threat like this since the 1918 Influenza. By the middle of March, many countries had implemented emergency measures, ranging from a complete lockdown to stay-at-home orders that put a curfew into effect. The US began to respond to the threat of COVID-19 with the enforcement of a lockdown toward the end of March, but the federalist structure of its government and the temperament of the political climate caused inconsistency and confusion in the response of the country.

When enforced properly, a lockdown can be one of the most effective non-pharmaceutical interventions applied within society [37]. A lockdown can prohibit disease spread by limiting social interaction to the necessity needed for a community's sustainability. In a strict lockdown, only essential businesses, like grocery stores and pharmacies, can remain accessible, whereas restaurants and entertainment venues should be closed to the public. These measures are estimated to have been effective in slowing the progression of COVID-19, especially when enforced before an observed increase in infection [38]. By handicapping the spread, lockdowns were able to give government officials more time to react to the incoming threat and healthcare providers more time to prepare for the anticipated influx of patients [39].

While lockdowns were useful, they could have been more effective if implemented earlier [38]. A study conducted in Spain even estimated that a nationwide lockdown imposed one day earlier would have lowered COVID-19 deaths within the country by 11 percent, and in turn, 23 percent of the 20,037 deaths could have been prevented by the end of the time period being analyzed [40]. Spain was of a few countries to implement a lockdown about one and a half months after its first case, but other countries, like the US and the United Kingdom (UK), waited about two months to implement them [39]. If their lockdown policy were enforced at an earlier date, many countries could have avoided such detrimental effects caused by COVID-19. Earlier lockdowns could have also been more effective if they had been paired with more sufficient duration and a progressive ending [39]. Spending more time in a restricted lockdown and easing restrictions more incrementally would have proven beneficial, as there would be a slimmer likelihood of resurgence [41].

The time at which a lockdown was implemented was not the only reason a lockdown was not as effective in the US; inconsistency between the decisions at the level of the state governments could also be to blame. At the beginning of the pandemic, the federal government cast the responsibility for the response to COVID-19 on the states, therefore, the US did not have a unified reaction like many other countries [42]. Some states responded with stay-at-home orders soon after the outbreak of the virus became significant, while others delayed the mandate for a time [42]. Though local initiatives were important, a cohesive response to this global health crisis enforced by a national government was proven to be more effective in other countries, like China [34].

The various containment strategies within each state reflected the political polarization found in the US as well [40]. Because the decisions concerning lockdown fell onto the government officials of the state, the political climate was a determinant in the implementation of a lockdown; states that primarily had a Republican-controlled governorship were less likely to mandate a lockdown in the earlier days of the pandemic [42]. The political officials were also primarily responsible for providing the policies to the public, which made partisanship a crucial factor when considering whether the guidelines were adhered to by the public [43]. For future responses to be more successful, bipartisan support should be considered as a way in which to increase adherence to government mandates.

The first of the 50 states to enact a shelter-in-place order was California. These lockdown measures are estimated to have led to between 160.9 to 194.7 per 100,000 population reduction in COVID-19 cases within the state as well as a 3.6 to 3.9 per 100,000 population reduction in COVID-19-related deaths per 100,000 [44]. Without these lockdown measures, California could have suffered larger losses, especially since it is a more densely populated state than most. Of the 50 states, Vermont was the state with the least incidences of COVID-19 cases [45]. Vermont was also among the first 21 states to implement a lockdown, asserting that an earlier lockdown could be beneficial in preventing the spread of COVID-19 [46]. Texas, New York, and Florida were some of the states with the largest numbers of cumulative cases of COVID-19 [47]. These statistics could be due to these states containing the most populous areas of the country, but it could also be attributed to its delay in mandating a lockdown. Overall, a more unified strategy between the states, such as a mandate from the national government, could have been more effective in preventing the spread of COVID-19.

Travel Screening

Out of the measures taken during the beginning of the COVID-19 pandemic, one that perhaps should have been better managed to prevent another public health failure in how the pandemic was handled by societies across the globe was the screening of travelers out of mainland China as well as contact tracing of who they encountered [48]. The measures that were taken at the beginning and during the pandemic included screenings done at borders and airports, as well as travel restrictions that were set in place to prevent unnecessary travel to areas with a high number of COVID-19 cases [48]. Additionally, travelers from COVID-19 hotspots were quarantined to avoid transmission [49]. However, due to the phenomenon of asymptomatic cases, it appears that screening for symptoms may not have been the best approach to screen travelers seeing as how individuals with no symptoms could still be carrying and spreading the disease [48]. Thus, a more effective approach to preventing the spread of the virus would have been for travelers to quarantine following any travel as well as receive PCR testing to confirm that they were not carrying the virus [49]. Because of the nature of the coronavirus, travel screening for domestic and international travel appeared to be ineffective in identifying positive cases [50]. This is because travel screening was primarily based on asking about symptoms and exposure [50]. This would mean that individuals who may have been asymptomatic could have been carrying the virus and answering “no” to screening questions based on symptoms; it becomes more difficult to determine exposure to the virus when a person can be contagious before ever developing symptoms or be asymptomatic altogether. Thus, this screening method appears to have faults. Furthermore, PCR testing in addition to this screening process is expected to be more effective but it is still not completely reliable because when a traveling individual is tested, they likely have not developed the findings that are consistent with the disease [50]. The final determination appears to be that quarantine, when followed for the appropriate period, is the most effective method of slowing or stopping the transmission of the virus. It is easy to see how enforcing quarantine for every traveler would have been quite difficult thus making a truly effective traveler screening process difficult to develop.

Hand Hygiene

Hand hygiene is a highly effective method of preventing the spread of pathogens and diseases that only became commonplace in the US a few decades ago, in the 1980s, when the firsthand hygiene guidelines made their debut into public health measures [51]. Today, hand washing and hand sanitizing are some of the most basic and accessible ways to maintain healthy communities by preventing disease and fighting against antibiotic resistance [52]. Following the COVID-19 pandemic, proper antiseptic techniques widely accessible to the community became crucial. COVID-19 is mainly transmitted through respiratory droplets and contact transmission, with touching being one of the primary ways in which an individual can become exposed to fluid infected with COVID-19 [53]. Thus, proper hand hygiene is key to preventing transmission.

For hand hygiene measures to be effective, they must be successfully enforced amongst the public and healthcare workers. Hand hygiene compliance is directly related to the accessibility and availability of resources that make proper hygiene possible. One of the most widespread methods of hand sanitizing available to the public became hand sanitizing stations available in drug stores, grocery stores, retail stores, schools, restaurants, and airports. Furthermore, handwashing stations were made available at places commonly visited by thousands of people. Most importantly, hand hygiene adherence was promoted amongst healthcare workers, who were most likely to encounter COVID-19. However, it seems that this was not entirely successful; a recent study showed that adherence to the hand hygiene guidelines established by WHO only significantly increased among healthcare.

Public health failed with the implementation of mask mandates because it was not implemented in a timely and uniform matter across the US. The states made the individual decisions of when to require the mask mandates and the specific regulations surrounding it. Some states failed to implement any mandates at all and left the local government to decide for themselves. States such as New York and Washington took on universal mask mandate policies, which showed a decrease in the spread of COVID-19 [10]. These universal mask mandates require residents of the state to wear masks whether indoor or outdoor areas regardless of close contact with other individuals. While masks proved to be an efficacious method to prevent COVID-19 transmission, there were many states which still had not implemented this policy on their residents. By September 2020, there were still 14 states which had not implemented any mask mandate orders [10]. To date, there are still 11 states which have never implemented mask mandates all over their population [11]. This can lead to a higher transmission rate among those states and prolong the spread of the virus. States such as Arizona, Utah, and Rhode Island had the highest cases per 100,000 people across the US [12]. All the previously mentioned states failed to issue a state-mandated mask order which led to a high increase in cases per capita [10]. While states such as Maryland, Oregon, and Pennsylvania quickly carried out the state-mandated mask order and ended up with some of the lowest averages in total cases [10].

Issues also became apparent with the type of masks used. The CDC recommended the use of N95 masks for healthcare workers, as they would have the highest exposure to the virus [9]. However, many civilians purchased these masks as well which led to a shortage in the healthcare field. This was a barrier many hospitals had to overcome by using surgical masks. The lack of information about the types of masks to be used had a substantial impact on the population because even though cloth and homemade masks were shown to be efficacious, the way they were fitted and used had a much larger impact. Surgical masks, which are meant for one-time use, were being reused by the public. These masks, when wet or dirty from constant reuse reduce the efficacy of preventing transmission [9]. Homemade and cloth masks were not always sized appropriately, and individuals had large gaps around the side of the masks or loose ear loops, which is directly contraindicated by the CDC [9].

Lastly, there was a shortage of personal protective equipment, specifically face masks, that the US was unprepared for when the pandemic hit. Increased use of surgical and N95 masks led to dwindling resources because the demand far exceeded the supply available [13]. Hospitals found themselves having to use reusable equipment when lacking the one-use equipment, they are usually afforded. Outside of the healthcare setting, there was a lack of masks provided to the public. While a state may have put in place a mask mandate, that does not inherently mean that a population has constant access to adequate masks. Only a few businesses were able to provide masks to their patrons, and this lack of availability led to individuals using inadequate replacements for masks such as neck gaiters, face shields, and masks with vents [14]. 

The timely and uniform execution of mask mandates, adequate supply, and properly informing the public are major considerations for future purposes. Several states showed much better results and decreased COVID-19 cases when those guidelines were followed [14]. Furthermore, healthcare workers could continue to perform their daily job duties without feeling at risk or having to quarantine themselves after a possible exposure. Leading more workers in the field to take care of those who become ill. Data showed that mask use is an efficacious way to prevent transmission of COVID-19 and proper application and supply can lead to a decrease in cases in the future [14].

## Conclusions

For future directions, the largest factor found that would lead to improvement in preventing the spread of COVID-19 is an earlier implementation of prevention methods. COVID-19 diagnostic methods also fell short and could have contributed to the spread of the disease. The first test that originally came out took about one week to deliver results, leaving the potentially COVID-positive patient to spread the virus to those around him/her. Once rapid testing began to be performed, which reduced the diagnosis time to two to three days, many turned out to be false positives or false negatives. These discrepancies in the testing process need to be addressed and more sensitive tests need to be provided to individuals who have had contact with COVID-19. While it is impossible to predict any future disease, this pandemic has a lot to teach about future preparations. Hospitals and healthcare facilities should remain stocked with personal protection equipment for worst-case scenarios to aid healthcare workers which will be facing new diseases. Healthcare facilities have many plans within their policies regarding major catastrophic events, with the experience they have had during COVID-19, they can use this as an opportunity to establish protocols and guidelines to enhance response to any future pandemics. Lastly, with the vaccine recently coming out, there was a lot of skepticism throughout the general population about taking the vaccine. The media and politics played a major role in deterring people from taking the vaccine. There was misinformation being spread about symptoms and possible uses of the vaccines which led the population to doubt their’ efficacy. Informing the public, instead of sensationalizing and profiting from such a challenging time would have created a better understanding for those with questions.
